# *Consent Builder*: an innovative tool for creating research informed consent documents

**DOI:** 10.1093/jamiaopen/ooac069

**Published:** 2022-07-27

**Authors:** Katherine A Sward, Rene Enriquez, Jeri Burr, Julie Ozier, Megan Roebuck, Carrie Elliott, J Michael Dean

**Affiliations:** Department of Nursing, University of Utah, Salt Lake City, Utah, USA; Department of Biomedical Informatics, University of Utah, Salt Lake City, Utah, USA; Department of Pediatrics, University of Utah, Salt Lake City, Utah, USA; Department of Pediatrics, University of Utah, Salt Lake City, Utah, USA; Human Research Protection Program, Vanderbilt University Medical Center, Nashville, Tennessee, USA; Duke Clinical Research Institute, Duke University Medical Center, Durham, North Carolina, USA; Duke Clinical Research Institute, Duke University Medical Center, Durham, North Carolina, USA; Department of Pediatrics, University of Utah, Salt Lake City, Utah, USA

**Keywords:** informed consent, user-centered design, IRB (ethics committees, research), multicenter studies

## Abstract

**Objective:**

To describe process innovations related to research informed consent documents, and development and formative evaluation of *Consent Builder*, a platform for generating consent documents for multicenter studies.

**Materials and Methods:**

Analysis of Institutional Review Board workflows and documents, followed by process redesign, document redesign, and software development. Locally developed software leverages REDCap and LaTeX. A small-scale usability study was conducted.

**Results:**

Process innovations were combining document types, and conceptualizing 2-part informed consent documents: part 1 standardizing the study description and part 2 with local site verbiage. *Consent Builder* was implemented in the Trial Innovation Network. User survey scores were acceptable; but areas for improvement were noted. LaTeX coding was the biggest challenge for users.

**Discussion:**

The process changes were generally well accepted. The software implementation uncovered un-accounted for assumptions, and variability in IRB review workflow across centers. Technical modifications may be needed before widespread implementation.

**Conclusion:**

We demonstrated proof-of-concept of an approach to generate research consent documents that are consistent across sites in study description, but which allow for customization of local site verbiage. The *Consent Builder* tool is an example of an operational innovation, helping meet a need that arose in part due to regulations around use of Single IRB for multicenter trials.

## INTRODUCTION

When the National Institutes of Health (NIH) issued the Single Institutional Review Board IRB (SIRB) policy^1^ in 2016, few investigators understood how informed consent documents in multicenter trials would be reviewed. The policy, updated in 2018,^2^ requires NIH-supported collaborative studies to use SIRB review. Collaborative, or *multicenter* studies, are conducted at multiple sites following the same study protocol.

SIRB use does not obviate the need for local review of the study and study documents. While the goal is to streamline Institutional Review Board (IRB) review, sites still need to comply with state and local policies, ensure that the research is feasible at their site, verify qualifications of local study staff, and conduct other Human Research Protection Program (HRPP) reviews. Uncertainty about how to implement SIRB is common^3^^,^^4^ and sometimes leads to duplicative full IRB review at study sites,^5^ with local edits subsequently introducing informed consent form (ICF) inconsistencies between sites.

The informed consent process is intended to help potential participants understand a study, including risks and benefits of the research and their rights as a participant. Researchers usually use a prewritten ICF, and participants sign and date the document to confirm that they have been provided the information and understand it. Informed consent documents are reviewed and approved by the Institutional Review Board(s) providing oversight for the study. Research nurses tend to rely on the ICFs^6^ typically reading the document verbatim to participants.^6^^,^^7^ Limitations to ICF documents are well known^8^ and although IRBs may provide templates or example text the informed consent documents can still fall short of desired readability^9^ leading to less than ideal participant understanding.^10^ Evolving regulatory and institutional requirements contribute to documents that are long and complex^11^ further contributing to understandability challenges.^12^ Guidelines were created for simplification of ICF language^9^ and the Revised Common Rule includes requirements that ICF documents begin with a concise presentation of key information^2^^,^^13^ although the best format for presenting this information still need to be studied.^14–16^ Research is beginning to demonstrate the value of augmenting standard ICF documents with multimedia,^12^^,^^17^ video, fact sheet, or other methods.^18^^,^^19^

There are situations where it may be important to modify consent documents from standard verbiage^20^ and there is evidence that document formatting can facilitate understanding^15^; however, in many cases sites request changes to consent documents that are merely preference and not substantive, or changes that focus on institutional interests (eg, liability management) rather than human research protections. Clear guidance is needed, including tools and templates, on how to comply with the SIRB policy. Platforms such as SMART IRB were designed to assist with regulatory aspects^5^ but tools and templates are needed to support creation and management of the informed consent documents.

There were multiple ways institutions deal with ICFs for multicenter research. Managing consent forms at the site is burdensome but generic documents may not meet the needs of participants.^21^ As SIRB regulation became active, document creation often shifted from local study teams to central coordinating centers^4^ but this created new challenges. The sheer number of documents can be overwhelming to coordinate and manage. Adult participants sign the ICF for their own participation. In pediatric studies parents sign a Parental Permission (PP) form for their child’s participation, and children who are old enough sign an Assent document. A multicenter trial at 20 sites enrolling children and young adults (a fairly typical scenario) would require 20 ICF, 20 PP, and 20 Assent documents. If the study is enrolling both English and Spanish speaking participants, the number of documents doubles. These documents must be kept in sync across sites when there are revisions or amendments.

The second challenge was how to support rigor and reproducibility for the consent process. Each site needs to operationalize the same study, yet has site-specific language as well. Local document creation causes multiple back-and-forth revisions between the coordinating center and participating sites.^4^ Standardized verbiage (“boilerplate language”) developed by local IRBs may provide portions of the site-specific language but each study still required customization.^22^^,^^23^

## OBJECTIVE

This article describes process innovations for informed consent documents, and the development and formative evaluation of software that implements the changes—a document building tool, *Consent Builder*. REDCap^24^^,^^25^ was already being used in some sites to create consent documents. The output was plain-text and additional formatting options were desired including lists and tables, and decorative elements such as dividing lines, logos, or precisely placed images.

## MATERIALS AND METHODS

The Utah Trial Innovation Center (TIC) is part of the Trial Network (TIN), formed in 2016 as an initiative within the national Clinical and Translational Science (CTSA) program. The TIN consists of the CTSA hub sites, 3 TICs, and a Recruitment Innovation Center (RIC). The TIN brings together decades of experience in conducting large-scale, multicenter clinical trials that recruit and engage challenging populations.^22^^,^^23^ IRB experts and experienced project managers at the TICs and RIC compared regulatory requirements, IRB checklists, and extant consent document templates to define the initial requirements and process innovations. IRB document review workflows and checklists guided initial software recommendations.

### Process innovations

#### Combined documents

Combining consent for research with HIPAA Authorization language is common practice at some centers. The adult ICF and Parent Permission (PP) forms use nearly identical verbiage. Two types of assent documents were common—a teen assent with verbiage similar to the ICF and PP forms, and a child assent with simpler language. Combining the ICF, PP, and teen assent into a single document was starting to be accepted at some sites.

#### Two-part consent documents

A breakthrough occurred with the conceptualization of consent forms as 2-part documents. Part 1 or General Language encompasses the study protocol and should be identical across all sites. This could be provided once by a central authority (the study PI). Part 2 (Local Language) includes local contact information and other site-specific verbiage.

### Technical innovation: *Consent Builder*

The Utah TIC developed a tool that implements the document process innovations. The goal was for project managers to be able to produce a richly formatted document that combined general study language (part 1) with site-specific language (part 2), formatted according to site-specific requirements and SIRB reliance agreements.^26^ Formatting preferences such as site logo or participant initials on each page needed to be able to be turned on or off separately for each study.

**Figure 1.**
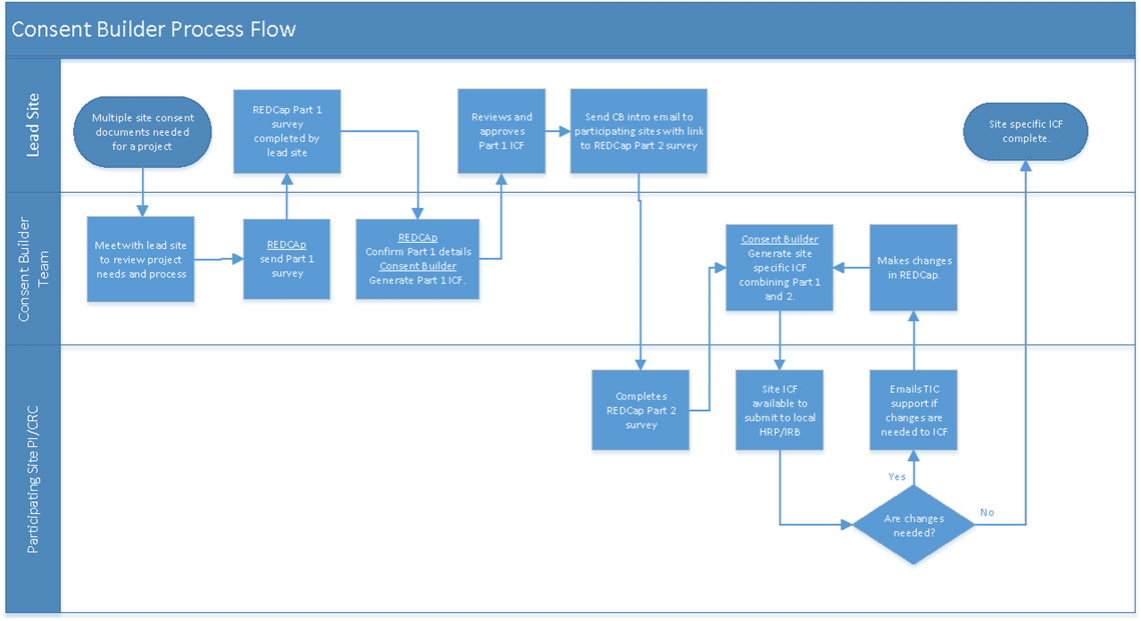
*Consent Builder* workflow. CB: *Consent Builder*; ICF: Informed Consent Form.

Figure 1 illustrates how a user interacts with the *Consent Builder* tools. The tool host site was the University of Utah. The host site sets up the study in the *Consent Builder* interface, and initiates REDCap surveys for the study. Multicenter studies designate a Lead Site (typically the site affiliated with the Contact PI of the study proposal). All other sites are considered Participating Sites.

1. **General language (the science)**: The Lead Site Principal Investigator or designee enters study protocol language into the Part 1 survey. The survey prompts for background information, study procedures, risks and benefits, and other content. Part 1 language may be separately reviewed/approved by the SIRB along with the initial study application if desired.
2. **Local language:** Participating sites are provided a copy of the formatted (and SIRB approved, if applicable) Part 1 document along with a link to the part 2 (Site) Survey. Study staff enter their site-specific language, and designate formatting preferences.
3. **Generate full consent document:** The host (coordinating center) staff use the *Consent Builder* tool to combine content from parts 1 and 2 and generate the formatted consent document, which is provided back to sites for review and submission to local IRBs.

### Architecture

**Figure 2.**
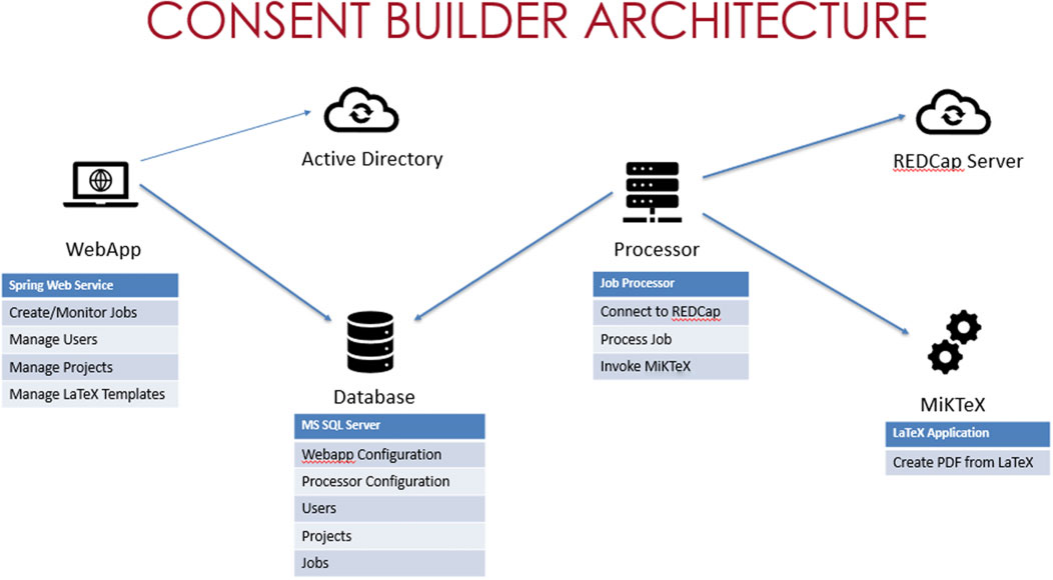
*Consent Builder* architecture.

*Consent Builder* is a suite of tools developed using a tiered web application/server architecture. The locally developed web application leverages REDCap^24^^,^^25^ for content management, LaTeX^27^ to encode formatting, and generates ICF in Adobe PDF^28^ format. The *Consent Builder* tool accesses survey content via the REDCap application programming interface (API), and merges this content with a LaTeX template stored in the *Consent Builder* application. Figure 2 depicts the logical division of *Consent Builder* components and functionality. The web application and service processor use the following components:

- Windows: Windows Server 2016 Datacenter
- Web Server: Apache Tomcat 8.5
- Java: AdoptOpenJDK
- TeX/LaTeX System: MiKTeX 2.9 or higher
- Directory Service: Microsoft Active Directory (AD)
- Database: MS SQL Server

The database server uses the following components:

- Windows: Windows Server 2016 Datacenter
- Directory Service: Microsoft Active Directory (AD)
- Database: MS SQL Server 2016

#### REDCap

Consent document content was collected and managed using the REDCap platform hosted at the University of Utah.^24^^,^^25^ REDCap (Research Electronic Data Capture) is a secure, web-based data capture platform, providing an intuitive interface; audit trails; automated export procedures; and procedures and an API for interoperability with external tools. The 3 TICs and RIC all utilized REDCap so it was a logical choice for initial content management. REDCap questionnaires are managed by the *Consent Builder* host site (University of Utah). An existing REDCap consent form template was modified for *Consent Builder* to support a 2-part document. Staff at the Utah TIC compared the part 1 and part 2 surveys with IRB review checklists and verified that required components were represented, and located in the appropriate survey part.

#### LaTeX

Prior experience suggested LaTeX^27^ as a document formatting platform that could accommodate all the formatting desired by investigators and IRBs. We selected LaTex in part because Utah staff had used the platform for many years to format study protocols. LaTeX is an open-source typesetting system designed for producing technical and scientific documents. It is widely used in fields such as physics or mathematics.^29^ Proponents embrace LaTeX because of the control it offers for complex document layouts, customizability, and portability, and it is readily compatible with many external programs. In *Consent Builder*, document components (headers) are defined in a LaTeX template, along with the ability to switch on or off optional features based on site survey responses. LaTeX provides this versatility via logic commands. Specific signature blocks can be created, and header verbiage adjusted. Images and logos can be added and their placement and dimensions dynamically controlled.

### User evaluation

Formative evaluation during development was via informal feedback. IRB experts at the TICs verified that the documents produced by the tool correspond to regulatory requirements. The tool and documents were reviewed at joint TIC/RIC meetings and individually. Regular meetings were held between the software developers, project managers, and IRB staff. During initial use, sites provided feedback directly to the developer. Quality Assurance included standard software code tests and user review. Project managers entered content from manually created documents into the surveys, generated new consent documents, and compared the original document to the newly generated document. IRB staff and experienced IRB reviewers evaluated the generated documents against IRB review checklists.

Perceptions from initial users about using the *Consent Builder* were formally evaluated in a survey that was administered online via REDCap. The University of Utah IRB determined the user study to be non-human subjects research (IRB number 00149404). We invited participation from project managers who had used the software to build consent documents for at least one actual study (not just testing). Usability and perceived utility were evaluated using the System Usability Scale (SUS).^30^ We selected the SUS as a short, validated instrument that is an industry standard^30–32^ and widely recognized benchmark that has been used to evaluate eConsent platforms.^33^ Limitations of the SUS are that it is minimalistic, originally designed for static interfaces, and may not capture complexities. We measured *perceived ease of use* and *perceived usefulness* using 2 questions from the Technology Acceptance Model (TAM) questionnaire^34^^,^^35^ and added custom questions specific to LaTeX. We continued to collect informal feedback, problems, and comments throughout the initial implementation.

## RESULTS

To date, 9 project managers have used *Consent Builder* to generate consent forms for 13 studies across 119 sites; generating more than 1238 documents. Document changes were largely acceptable to site IRBs although a few were unwilling to allow certain changes such as including HIPAA authorization language within the ICF document. There were site differences in whether part 1 and part 2 could be combined or needed to be reviewed separately. We received survey responses from 7 of the 9 project managers. Experience with the tool varied from use with 1–15 studies. SUS scores range from 0 to 100; and are interpreted as normalized scores (not a percentage).^32^ The overall SUS score was 74.58; a score that is considered *acceptable*^30^ but with room for improvement. Table 1 shows the responses to other questions. LaTeX coding problems were the most frequent cause of errors in generating PDF documents.

**Table 1.** Learnability and perceived utility

Question	Avg score^a^
I needed to learn a lot of things before I could get going with *Consent Builder*	2.5
I needed to learn a lot of things to start to use LaTeX code within *Consent Builder*	3.5
I needed to learn a lot of things other than LaTeX, to get going using *Consent Builder*	2.2
Using *Consent Builder* in my job would enable me to accomplish tasks more quickly	4.3
I would find *Consent Builder* useful in my job	4.3

a Scored 1 = strongly disagree to 5 = strongly agree.

## DISCUSSION

### Lessons learned/next steps: software

Although the *Consent Builder* tool had acceptable usability metrics, and we expect that the principle of 2-part ICF documents will likely gain acceptance, refinement of the *Consent Builder* tool is needed before hosting could be done at other sites.

Sites responding to surveys do not need to have REDCap installed, because the surveys are accessed in a web browser. However, the choice of REDCap as a content database constrains hosting to sites that have local installations of REDcap. Other databases could manage content but would require modifications to the API calls. In addition, we had manually compared site templates to develop the content questionnaires. A formal data model and standardized representation of for informed consent document content would support more rigorous quality assessment.

The most pressing challenge was use of LaTeX for content formatting. Although LaTeX had been in long use by Utah staff, other personnel including site staff needed to learn LaTeX coding. Challenges with using LaTeX have been reported even by experienced users.^36^ We are exploring options to address this challenge. As an interim option, we have assigned LaTeX coding to a centralized expert, who inserts formatting code after sites enter content verbiage into REDCap. Although this reduces burden on site staff, central coding can cause a workflow bottleneck. An alternative may be to incorporate a graphical LaTeX editor, which would make the coding easier for novice LaTex users. We are considering replacing LaTeX with a different formatting markup, although options explored to date such as HTML or rich text format formatting similarly require coding to accommodate the formatting desired by sites.

### Lessons learned/next steps: research culture

User preconceptions were an unexpected challenge, including lack of recognition that survey responses would be copied verbatim into the consent document. For example, some users simply typed a name and phone number, assuming the system would automatically convert this to a full paragraph of local contact information text. More detailed user documentation and a user training video^37^ were developed to help manage expectations about how the system works, and exemplar response verbiage was added to the REDcap surveys.

The *Consent Builder* documents aligned well with University of Utah SIRB workflow. The University of Utah IRB embraced the principle of a 2-part consent document, and will accept PDF documents for review. This is not the case at all sites. Efficiencies can be gained by standardizing documents, but users continue to request changes to documents. Many of those requests are preference-based and not substantive. The culture of every researcher wanting a bespoke consent form needs to change along with the processes we use.

*Consent Builder* addresses a different problem than electronic document signature (eConsent) platforms. Electronic platforms can support participant understanding, at least for some aspects of a study^38^ by integrating avatars, on demand glossary, videos, or other features.^39^ We recognize that as eConsent tools mature the needs that drove *Consent Builder* development might be met through other tools. At present, however, centers that coordinate multicenter trials continue to express interest in a tool like *Consent Builder* that can aid in managing informed consent documents.

## CONCLUSION

Innovations are new products, ideas, methods, or processes.^40^ The TICs and RIC focus on *operational innovations*^41^ which are improvements in how studies are designed or conducted.^42^^,^^43^ Combined ICF reduce the number of documents that need to be managed. Two-part consent keeps the study description consistent across sites. We demonstrated initial proof-of-concept of a *Consent Builder* tool, which meets a need to support centralized management of the ICF documents in a multicenter trial.

Tools like *Consent Builder*, or templates within eConsent systems, primarily standardize document structure and format. There continues to be a need for research regarding the *content* of those documents. Improving informed consent will require evolution in the culture around informed consent documents. Improved understanding of the regulations may support reduced document complexity. Further research may provide evidence that changes from a culture of customized documents based on personal preference, to customizations that are evidence based.

## FUNDING

This work was supported the National Center for Advancing Translational Sciences of the National Institutes of Health (NIH/NCATS) grant numbers U24TR001597, U24TR001608, U24TR001597, and U24TR001609.

## AUTHOR CONTRIBUTIONS

All listed authors meet the criteria for authorship. JB and JMD focused on tool conception and process improvements. JO, MR, and CE analyzed IRB workflows, evaluation of document changes, and tool output fit with IRB processes. RE was the initial architect and software developer, and collected data for the evaluations. KAS contributed to software design, conception and design of the user study, IRB approval of the user study. ER, JB, and KAS provided the initial paper content. All authors critically revised content, provided final approval of the version to be published, and agree to be accountable for all aspects of the work.

## Data Availability

Data from the user survey may be obtained upon request from the corresponding author. *Consent Builder* software intellectual property is managed by the University of Utah Partners for Innovation, Ventures, Outreach, and Technology (PIVOT) center.
